# Barriers to Sustainable Telemedicine Implementation in Ethiopia: A Systematic Review

**DOI:** 10.1089/tmr.2020.0002

**Published:** 2020-11-18

**Authors:** Getu Gamo Sagaro, Gopi Battineni, Francesco Amenta

**Affiliations:** ^1^ Telemedicine and Telepharmacy Center, School of Medicinal and Health Products Sciences, University of Camerino, Camerino, Italy.; ^2^ Research Department, International Radio Medical Center (C.I.R.M.), Rome, Italy.

**Keywords:** telemedicine, telehealth, m-health, e-health, barriers

## Abstract

**Background:** Different studies showed that the use of telemedicine is effective in reducing hospital burden, suffering from patients, need of transports, hospital fear, save money and time, and increasing the quality of health care. However, the implementation of telemedicine countenances different challenges in developing countries generally and in Ethiopia, particularly. This review aims to evaluate barriers affecting sustainable telemedicine implementation in Ethiopia.

**Methods:** PubMed (Medline), Google Scholar, Embase, and Scopus databases were searched between July 4, 2020 and July 28, 2020. Studies published between 2005 and June 30, 2020 were considered. Relevant articles were selected by reviewing keywords, titles, and abstracts. Out of 40 articles, 33 articles remained after removing duplicates. We finally analyzed 14 articles from the mentioned databases based on our eligibility criteria and identified different barriers. We followed the preferred reporting items for systematic review and meta-analyses (PRSIMA 2009) checklist for this review.

**Results:** We identified 25 barriers through 14 articles and classified barriers into organizational, users, and staff and programmers' barriers. Accordingly, organizational, users, and staff and programmer barriers were 12 (48%), 7 (28%), and 6 (24%), respectively, with the frequency of occurrence through 14 articles. Cost, awareness, and resistance to change were the most frequently reported barriers among organizational, user, and staff and programmer barriers, respectively.

**Conclusions:** Infrastructure and costs were the most frequently reported barriers, and staff resistance to change was also the critical factor in influencing the sustainable implementation of telemedicine in Ethiopia.

## Introduction

Telemedicine is defined as exchanging health information or providing health care services through electronic communication and information technologies when actors are in different locations. We used the WHO (World Health Organization) definition of telemedicine: “The provision of health services, where distance is a critical factor, by all health professionals who use information communication technologies (ICT) for valid exchange information for the diagnosis, treatment, and prevention of diseases and injuries, research and evaluation and for continuing education of health care providers, all in the interest of advancing the health of individuals and their communities.”^1^ As per the WHO, which does not distinguish between telemedicine and telehealth, we used the word telemedicine to refer to telemedicine or telehealth for this systematic review.^1^

Telemedicine can provide a significant contribution to the health industry in developed countries and allows the following: (1) real-time communication connects patients with health care providers through videoconferences, home-health monitoring devices, and telephone at anytime and anywhere; (2) store and forward refers to share images, data, voice, video, and other medical information with physicians for diagnosis across a long distance; and (3) remote patient monitoring to monitor patient health parameters and collect various health-related data while clients are at home and submit their health information for monitoring, evaluation, and response.^1,2^ Moreover, telemedicine plays a critical role in solving the health care access problem in remotely located patients. This is true primarily in the maritime environment where telemedicine allows health care and advice for seafarers.^3–7^ Telemedicine is also expected in developing countries to reduce hospitals' burdens, suffer from patients, need for transports, hospital fear, save money and time, and increase the quality of health care.^8^ However, telemedicine's implementation remains difficult in developing countries, and studies have shown that almost 90% of telemedicine projects have been abandoned or failed in developing countries.^8,9^

This study reviews telemedicine's present status in Ethiopia and discusses barriers to sustainable implementation. Globally, very little review has been done on barriers to the uptake of telemedicine.^10^ In most sub-Saharan African countries, especially in Ethiopia, there are different challenges related to telemedicine diffusions, such as ICT policies, intersectoral collaboration, laws and regulations regarding the use of e-health, strategies that facilitate telemedicine services, and integrated supportive supervision on telehealth adoption, and ICT equipment. These challenges differ from region to region as well as between urban and rural areas. However, so far, no review has examined the barriers to telemedicine's sustainable implementation in Ethiopia. This is the first review reporting the barriers or challenges to telemedicine diffusion in Ethiopia.

Ethiopia is one of the East Africa countries and located in Africa's horn, bordered by Kenya, South Sudan, Sudan, Somalia, Djibouti, and Eritrea. Ethiopia has an estimated total population of 110,175,175 (as of Thursday, July 11, 2019, as stated by the latest United Nations estimates), which ranks 12th globally and the most populous country in Africa after Nigeria. Ethiopia's population density is 115 people per km^2^ (298 people per mi^2^); the total land area is 1 million km^2^ (386,102 mi^2^).^11^ Besides, 79% of the total population live in rural areas (87,038,388 people in 2019).^11^ The median age in Ethiopia was approximately 18.8 years and the life expectancy at birth for women and men was reported 65 and 62, respectively, in 2019. The neonatal, infant, and <5 years death rates are 29, 48, and 67 per 1000 live births, respectively, and the maternal mortality rate is 412 per 100,000 women in 2016.^12^

Sixteen years ago, Ethiopia started expanding access to primary health care through community health extension programs and health centers.^13^ Despite this, health care service access is a problem, especially in remote or rural areas, because of the high population growth rate, increased demand for health care, slow economic growth, and rising health costs. An additional problem is the shortage of medical specialists. This requires that patients travel long distances where specialists are located. In contrast, the waiting time for treatment after arriving may take more than a week. Inadequate transportation and hard-to-reach geographical locations make access to health care services quite difficult in Ethiopia, where >79% of the population live in rural areas. An option to allow access to health care services in a developing country such as Ethiopia is implementing telemedicine services and ensuring sustainability.

Ethiopia adopted a national e-health strategy in 2014 and identified five main areas for e-health implementation^14^: (1) health information system, (2) telemedicine, (3) m-health, (4) e-learning (health workforce training), and (5) community information. However, e-learning, especially health information technicians (HITs) training and allocating at health centers and hospitals, is promising but still too young. Some of the other strategies are occasionally implemented, and others are not implemented yet in practice. This review aimed to evaluate the factors affecting telemedicine's sustainable implementation in Ethiopia by analyzing the published studies from 2005 to June 30, 2020. The result of this review could help authorities to understand different bottlenecks in telemedicine implementation, to prioritize the problems, and to take appropriate measures.

## Methods

### Searching strategy

Different electronic databases were searched to identify the relevant studies, that is, *PubMed Central (Medline), Google Scholar, Embase*, *and Scopus*. Searches of the internet were carried out using different search engines and performed between July 4, 2020, and July 28, 2020. The keywords used for searching in this review were “Barriers” OR “Challenges” AND “Implementation” OR “Adoption” AND “Telemedicine” OR “e-Health” OR “mHealth” OR “telehealth” OR “mobile health” AND “Ethiopia.” Additional articles were also manually extracted from the reference list of selected articles to obtain relevant information on the barriers to telemedicine services in Ethiopia. Initially, we used keywords, abstracts, and titles. Finally, we have selected the relevant articles by reviewing the full text of the articles.

### Inclusion and exclusion criteria

Studies are expected to describe the barriers to telemedicine or telehealth services in Ethiopia to be considered in this review. The eligibility criteria for selecting studies included peer-reviewed journals and articles between 2005 and June 30, 2020, studies published in English, full text, descriptive, cross-sectional, and mixed-method studies reporting barriers to implementing telemedicine in Ethiopia. The mixed-method study is one of the research study designs considering both quantitative and qualitative methods. In contrast, studies published only as abstracts were excluded. We also included interventional research and narrative analysis, which gave information on the implementation of telemedicine and telehealth in Ethiopia. The keywords, title, and abstracts were assessed independently by three (G.G.S., G.B., and F.A.) reviewers.

### Quality assessment

The critical appraisal tool for descriptive and cross-sectional studies was used to evaluate the quality of selected studies. The critical appraisal tool has 11 questions adapted from Guyatt et al.,^15^ and the questions are broadly categorized into three parts: (1) Are the study results valid? (2) What are the results? (3) Will the results help locally? (Supplementary Table S1).

### Data extraction

The following data were taken from selected studies: (1) name of the first author, (2) publication year, (3) study design, and (4) types of barriers. Microsoft (MS) Excel spreadsheets were then used to enter the extracted data. Accordingly, we identified different barriers that affect telemedicine and telehealth in Ethiopia and organized extracted data into a table.

## Results

### Relevant articles

**FIG. 1.**
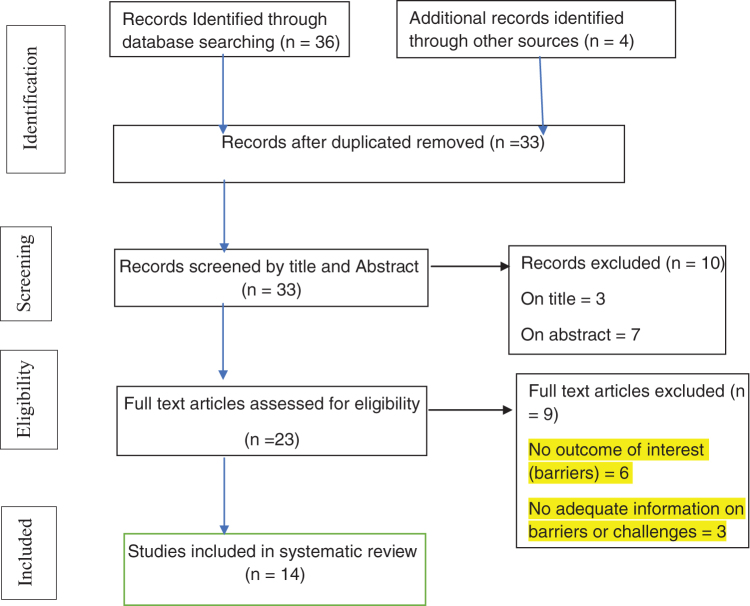
PRISMA flow chart of literature showing the search process, the reasons for exclusion of studies, and the final number of articles included in the review.

Overall, 40 potentially relevant articles were identified, and the studies were filtered using publication years ranging from the year 2005 to June 30, 2020. Out of 40 potentially relevant articles, 33 studies remained after the removal of 7 duplicates. The selection of abstracts and titles rejected 10 studies. The full article was retrieved after the reviewers' approval based on the selection criteria, and the entire article was again assessed separately, and nine studies were excluded at this stage. Finally, based on our inclusion criteria, 14 articles were selected for a systematic literature review, and three (G.G.S., G.B., and F.A.) reviewers read each article and made notes to identify barriers (Fig. 1).

We identified 25 different barriers by reviewing 14 selected articles, and the summary of the results is presented in Table 1.

**Table 1.** The Selected Articles Are Listed in the Table with the First Author Name, Study Design, and Types of Barriers in Ascending Order of Publication Years

Refs.	Publication year	Study design	Barriers
Kifle et al.^17^	2006	Mixed method	Costs, electricity, ICT infrastructures, human resources, language, culture, educational level, readiness, awareness, policies, technical support system, reimbursement, bandwidth of dwelling, resistance to change
Shiferaw and Zolfo^18^	2012	Descriptive case report	Cost, e-health policy, implementation model/guideline, ICT infrastructure, language, awareness, socioeconomic status, culture, internet connection, the bandwidth of dwelling
Little et al.^20^	2013	Cross-sectional	Cost, technical support, internet connection
Mengesha et al.^27^	2013	Case study	An internet connection and technical support system
Abera et al.^21^	2014	Mixed method	Cost, availability of technical support, Internet connection, ICT infrastructure, technological infrastructure, readiness, staff turnover
Medhanyie et al.^13^	2015	Cross-sectional	Health system, resistance to change, ICT infrastructure, awareness, language, poor application design, and technically challenged staff.
Xue et al.^16^	2015	Cross-sectional	The technical support system, resistance to change, anxiety, costs
Dusabe-Richards et al.^19^	2016	Mixed-method	Network coverage, technical support system, language, phone (mobile), socioeconomic status
Barkman and Weinehall^22^	2017	Descriptive case report	Reimbursement, infrastructures, cost
Biruk and Abetu^32^	2018	Cross-sectional	awareness
Steege et al.^23^	2018		Costs, level of education, culture, phone (mobile), awareness
Shiferaw et al.^24^	2018	Cross-sectional	Cost, health system, culture, staff turnover, ICT infrastructure, electricity, phone (mobile), service integration, internet, awareness
Harding et al.^25^	2019	Descriptive case report	Electricity, e-health literacy, health system, telecommunication service, cost, internet connection, phone (mobile), human resources
Dessie Gashu et al.^26^	2020	RCT	ICT infrastructure, e-health literacy, phone (smart mobile)

ICT, information communication technologies; RCT, randomized controlled trial.

The reported 25 barriers were categorized into user barriers, organizational barriers, and staff and programmer barriers.

### User barriers

As given in Table 2, seven various users' (clients/patients) barriers were identified by reviewing different literature during the study period. These barriers included a limit of computer or e-health literacy, the bandwidth of dwelling, access to the mobile phone, level of education, socioeconomic factors, cultural factors, and unawareness of telehealth products' existence and services. Hence, awareness was the most frequently mentioned consumer barrier represented by 26% (6/23) among user barriers. Phone (smart mobile) access 22% (5/23) and cultural factors 17.4% (4/23) were the second and third often reported barriers of telemedicine implementation in Ethiopia.

**Table 2.** User Barriers Reported in Selected Articles from 2005 to 2020

User barriers	Frequency (%)
Awareness	6 (26)
Phone (mobile)	5 (22)
Culture	4 (17.4)
Bandwidth of dwelling	2 (8.7)
Level of education	2 (8.7)
Socioeconomic status	2 (8.7)
e-Health literacy	2 (8.7)

The consumer's educational level has a high impact on the acceptance of telemedicine services, and the socioeconomic status influences access to a cell phone in Ethiopia. The cultural factors also affect the adoption of telehealth services because there is still a challenge in rural communities related to women's health care decisions. They do not decide alone about their health care and use e-health services even to participate in awareness programs, including meeting and discussion without permission. This is true for married women. Cultural factor is the main challenge to adopt telehealth services in a remote area, and it will cause a lack of confidence in consumers, and then it to be challenging to convince them to receive health care services without visiting a health care professional (Table 2).

### Organizational barriers

Overall, 12 main types of organizational barriers were selected in the accepted articles (Table 2). Consequently, the cost was the most frequently reported barrier to telemedicine implementation and accounted for 20.5% (9/44) of organizational barriers. ICT infrastructure 18.2% (8/44), internet connection 13.6% (6/44), technical support system 13.6% (6/44), electricity 6.8% (3/44), health system 6.8% (3/44), and human resources 4.5% (2/44) were the most common organizational barriers to the sustainable implementation of telemedicine services in Ethiopia (Table 3).

**Table 3.** Organizational Barriers Reported in Accepted Articles from 2005 to 2020

Organization barriers	Frequency (%)
Cost	9 (20.5)
ICT infrastructure	8 (18.2)
Internet connection	6 (13.6)
Technical support system	6 (13.6)
Health system	3 (6.8)
Electricity	3 (6.8)
Human resources	2 (4.5)
Reimbursement regulations	2 (4.5)
Staff turnover	2 (4.5)
Implementation model/guideline	1 (2.3)
e-Health policy	1 (2.3)
Service integration	1 (2.3)

### Staff and programmer barriers

We identified six main types of staff and programmer barriers by reviewing the selected articles, which account for 24% (6/25) of all described barriers. These barriers included technically challenged staff, resistance to change, readiness, language problems, poor design and anxiety. The health care professional's resistance to telemedicine adoption in Ethiopia was the main influencing factor for successful service implementation. If physicians consider that telemedicine would reduce autonomy and undermine their privileges, the result of this technology would be affected. After appraising losses of some incentives and considering their professional autonomy, health professionals perceived telemedicine as a threat. According to some studies, health care professionals tend to become nervous when they are requested to use telemedicine, and their anxiety results in a negative attitude about telemedicine services, and they are likely to engage in resistance.^16^ The resistance of health professionals to change is perhaps due to the lack of a reliable reimbursement system for providing health care through telemedicine in Ethiopia. Providers who invested in telemedicine were committed to reimbursement and taking a return on their investment.^10^ In contrast, telemedicine service implementation requires significant changes to the existing workflow; staff and providers must invest their time by training new workflows and techniques. This may influence the efficiency and effectiveness of the successful telemedicine service establishment.

Medhanyie et al.^13^ and Xue et al.^16^ reported that technically challenged staff members were critical barriers next to resistance to change to the successful implementation of telemedicine and ensure the sustainability of services in developing countries including Ethiopia. Moreover, four studies reported that language limitation was another barrier affecting users' friendly operating system.^13,17,18,19^ This implies that the consumer loses confidence in adopting telehealth services at the local level (Table 4).

**Table 4.** Staff and Programmer Barriers Reported in Selected Articles from 2005 to 2020

Staff and programmer barriers	Frequency (%)
Resistance to change	4 (33.3)
Language	3 (25)
Readiness	2 (16.7)
Design/system	1 (8.3)
Anxiety	1 (8.3)
Technically challenged staff	1 (8.3)

### Methodological quality assessment of the selected studies

The appraisal of the methodological quality of the identified studies is presented in Supplementary Table S2. The 14 studies were selected after a critical appraisal. Accordingly, in three studies, data collection tool validation was not mentioned, and in two studies, the sample size calculation was not included. The remaining nine studies had good methodological quality (Supplementary Table S2).

## Discussion

### Summary of main findings

According to this review, the most frequently reported barriers to the implementation of telemedicine services are cost^17,18,20,21,16,22,23–25^ and infrastructure.^13,17,18,21,22,24,26^ The initial cost of establishing telemedicine services is very high, and to function telemedicine services in rural or remote areas for underserved communities needs telecommunication expenses, training (health personnel and patients), needs for new advanced technologies, and electric power supplies. Fifteen years ago, in Ethiopia, all concerned bodies such as universities, telecommunication authority, the ministry of health, policy makers, and other responsible institutions agreed to support telemedicine implementation.^21^ However, some services such as access to electricity power (only 8% of rural household have access in 2016),^12^ e-health literacy, access to health information technologies (IT) to address gaps in IT literacy for health extension workers,^25^ network coverage, and internet connectivity,^18,20,21,24,25,27^ and HITs were still the main bottlenecks for the implementation of telemedicine services in Ethiopia. We recommend that the Ethiopian government focus on organizational barriers, especially infrastructure challenges such as unstable power supplies, poor communication networks, inadequate internet connectivity with limited bandwidth, lack of human resources with necessary technical expertise to successfully implement telemedicine, and ensure its sustainability. Most health care institutions, telecommunication, and electricity infrastructures are owned and controlled by the government. Moreover, the government should invite other stakeholders such as nongovernmental organizations to support technical issues and financial resources at the initial phase.

In contrast, culture^17,18,23,24^ and staff resistance to change^13,17,16^ are critical factors affecting telemedicine implementation in developing multicultural countries such as Ethiopia. The adoption of telemedicine services requires the acceptance of users involved in the process. This review may indicate that the unawareness or lack of comfort in the use of telemedicine services because the willingness of change and adherence to new services is dependent on the culture of users, organizations, and providers (employees). Many health care providers, especially physicians, consider telemedicine to make physician–patient communication ineffective, and telemedicine implementation may alter their current work practices.^16,28,29^ This negative perception leads health professionals to resistance to telemedicine adoption.

Furthermore, health care professionals' readiness to provide this modality is another important factor influencing telemedicine service diffusions. As a result, national and local level advocacy on the benefit and proper use of telemedicine services and individual training would help address fears and resistance and increase acceptance among health professionals and users or patients correspondingly. Reimbursement regulations and other rules/laws regarding telemedicine services should be strengthened and enforced to encourage health care professionals to participate in telemedicine care modalities. From the users' perspective, rural people sometimes struggle to believe that they can get health services without having face-to-face communication. This could be a lack of awareness of the potential benefits of telemedicine services. Therefore, efforts such as training and continuous education for health care providers and their communities could reduce the limitation and increase awareness of receiving health care through this modality. Governments, community leaders, delegates, and elders should take responsibility for changing cultural behaviors as well as misunderstandings about telemedicine services through public education. Socioeconomic status is one of the user's barriers to influence the diffusion of this technology by limiting access to the smartphone (mobile). As a result, Ethiopia's mobile subscription rate was 8% in 2017, which was very low compared with other African countries such as Kenya (51%), Uganda (30%), and Tanzania (31%).^30^

This review reported that staff turnover was another critical factor in influencing the spread of telemedicine. Ethiopia is one of the African countries with a high turnover of personnel, particularly health workers, and the loss of skilled personnel can affect customization of service and implementation of technology.^21,24,31^ Hence, the government and other responsible bodies should retain qualified personnel by providing advancement opportunities such as career advancement and incentives and encouraging other workplace motivations.

Language is also another barrier^13,17,18,19^ because users have no technical support; limited language knowledge is another barrier for the system to be user friendly. Ethiopia is a multilingual country; therefore, considering at least the national language during design phase is essential for overcoming this barrier to successful implementation and enabling the diffusion of telemedicine services. A technical solution should be considered related to language issues at the programmer level. Sometimes users and providers encounter the technology itself to be a challenge,^13,25^ because some users or providers may not be as familiar with the functionality of their mobile devices, internet connection, or telemedicine applications. It would be crucial that technical supporters offer guidance for successful implementation and ensure users' confidence at the local level. As for this review's limitation, we searched four well-known electronic databases for relevant articles and manually retrieved articles from the reference list of published articles, but we have limited studies regarding telemedicine services in Ethiopia. In fact, telemedicine is an understudied field and a poorly applied medical modality in Ethiopia. These could be the reasons for the limitation of studies on this technology. More than one reviewer examined each article to avoid selection bias, but we did not control for publication bias. Overall, nine studies have good methodological quality, but the remaining five studies have low quality due to data collection tool validation and sample size issues. However, this review provides factual information on the barriers or challenges related to telemedicine adoption in Ethiopia.

## Conclusions

Infrastructure and cost were the most frequently reported barriers in this review. In other words, ∼85% of the selected articles reported that cost and infrastructure were the critical barriers to telemedicine implementation in Ethiopia. Furthermore, staff resistance to change and staff turnover have also been reported to influence telemedicine's successful implementation. Inadequate availability of infrastructure, especially ICT infrastructures such as computers, power supply, internet connection, and inadequate HITs, could be overcome through training, repair and replacement, budget allocation, and the purchase of new technologies, improving access to power, and network and internet connection. Initial cost for establishment, telecommunication expenses, cost for training service providers, and patients' expenses could be covered by the government and by other stakeholders such as nongovernmental organizations through the government. In this respect, the Federal Ministry of Health and telecommunication authority should work closely with each other to integrate health information with ICT development. Ethiopia Federal Minister of Health (FMoH) should give attention to policy and e-health strategies to outline the visions and objectives regarding applications, provisions, control, and standards related to telemedicine solutions and focus on integrating e-health strategies with national health policy. Incorporating e-health strategies with national health policy could facilitate and increase the chances of successful and sustainable implementation of this technology by providing a structural frame and protocol for planning and developing services. In general, this study provides directions to the government to prioritize the problems and develop appropriate solutions.

## Supplementary Material

Supplemental data

Supplemental data

## Abbreviations Used

HIT: health information technician
ICT: information communication technologies
IT: information technologies
WHO: World Health Organization
