# Supplementary material for: Barriers to Sustainable Telemedicine Implementation in Ethiopia: A Systematic Review
Source: Telemed Rep. 2020 Nov 18;1(1):8–15. doi: 10.1089/tmr.2020.0002 (PMC8812291; doi:10.1089/tmr.2020.0002)
Supplement: Supplemental data [file Supp_TableS1.docx]

**Supplementary Table S1. Methodological quality assessment tool screening questions for descriptive/cross-sectional studies**

| Q.no | Screening questions |  |  |  |
| --- | --- | --- | --- | --- |
| 1 | **Did the study address a clearly focused issue?** | Yes | No | Can’t Tell |
|  | HINT: A question can be focused in terms of:  – the population(s) studied  – the health measure(s) studied (e.g., risk factor, preventive behavior, outcome) |  |  |  |
| 2 | **Did the authors use an appropriate method to answer their questions?** | Yes | No | Can’t Tell |
|  | HINT: Consider  - Is a descriptive/cross-sectional study an appropriate way of answering the question?  - Did it address the study question? |  |  |  |
| 3 | **Were the subjects recruited in an acceptable way?** | Yes | No | Can’t Tell |
|  | HINT: We are looking for selection bias which might compromise the generalizability of the findings:  - Was the sample representative of a defined population?  - Was everybody included who should have been included? |  |  |  |
| 4 | **Were the measures accurately measured to reduce bias?** | Yes | No | Can’t Tell |
|  | HINT: We are looking for measurement or classification bias:  - Did they use subjective or objective measurements?  - Do the measures truly reflect what you want them to (have they been validated)? |  |  |  |
| 5 | **Were the data collected in a way that addressed the research issue?** | Yes | No | Can’t Tell |
|  | Consider:  – if the setting for data collection was justified  – if it is clear how data were collected (e.g., interview, questionnaire, chart review)  – if the researcher has justified the methods chosen  – if the researcher has made the methods explicit (e.g., for the interview method, is there an indication of  how interviews were conducted?) |  |  |  |
| 6 | **Did the study have enough participants to minimize the play of chance?** | Yes | No | Can’t Tell |
|  | Consider:  – if the result is precise enough to make a decision  – if there is a power calculation. This will estimate how many subjects are needed to produce a  reliable estimate of the measure(s) of interest. |  |  |  |
| 7 | **How are the results presented, and what is the main result?** | Yes | No | Can’t Tell |
|  | Consider:  – if, for example, the results are presented as a proportion of people experiencing an outcome, such  as risks, or as a measurement, such as mean or median differences, or as survival curves and hazards  – how large this size of the result is and how meaningful it is  – how you would sum up the bottom-line result of the trial in one sentence |  |  |  |
| 8 | **Was the data analysis sufficiently rigorous?** | Yes | No | Can’t Tell |
|  | Consider:  – if there is an in-depth description of the analysis process  – if sufficient data are presented to support the findings |  |  |  |
| 9 | Is there a clear statement of findings? | Yes | No | Can’t Tell |
|  | Consider:  – if the findings are explicit  – if there is an adequate discussion of the evidence  both for and against the researchers’ arguments  – if the researcher has discussed the credibility of their findings  – if the findings are discussed in relation to the original research questions |  |  |  |
| 10 | Can the results be applied to the local population? | Yes | No | Can’t Tell |
|  | HINT: Consider whether  - The subjects covered in the study could be sufficiently different from your population to cause  concern.  - Your local setting is likely to differ much from that of the study |  |  |  |
| 11 | How valuable is the research? | write comments here | | |
|  | Consider:  – if the researcher discusses the contribution the study makes to existing knowledge(e.g., do they  consider the findings in relation to current practice or policy, or relevant research-based literature?)  –if the researchers have discussed whether or how the findings can be transferred to other  Populations |  | | |
