# Supplementary material for: Barriers to Sustainable Telemedicine Implementation in Ethiopia: A Systematic Review
Source: Telemed Rep. 2020 Nov 18;1(1):8–15. doi: 10.1089/tmr.2020.0002 (PMC8812291; doi:10.1089/tmr.2020.0002)
Supplement: Supplemental data [file Supp_TableS2.docx]

**Supplementary Table S2. Methodological quality assessment of the selected studies**

| **Study ID** | **Q1** | **Q2** | **Q3** | **Q4** | **Q5** | **Q6** | **Q7** | **Q8** | **Q9** | **Q10** | **Q11** |
| --- | --- | --- | --- | --- | --- | --- | --- | --- | --- | --- | --- |
| Kifle M et al | yes | yes | yes | yes | yes | yes | yes | yes | yes | yes | good |
| Shiferaw F et al | yes | yes | yes | Can’t tell:  questionnaire validation not included | yes | yes | yes | yes | yes | yes | good |
| Little A | yes | yes | yes | yes | yes | Can’t tell:  Sample size calculation not included | yes | yes | yes | yes | good |
| Mengesha GH et al | yes | yes | yes | Can’t tell:  questionnaire validation not included |  | Can’t tell: | yes | yes | yes | yes | good |
| Abera, A. A et al | yes | yes | yes | yes | yes | yes | yes | yes | yes | yes | good |
| Medhanyie AA et al | yes | yes | yes | yes | yes | yes | yes | yes | yes | yes | good |
| Xue Y et al | yes | yes | yes | yes | yes | Can’t tell:  Sample size calculation not included | yes | yes | yes | yes | good |
| Dusabe-Richards JN et al | yes | yes | yes | Can’t tell:  questionnaire validation not included | yes | No:  convenient sampling | yes | yes | yes | yes | good |
| Barkman C et al | yes | yes | yes | Can’t tell | yes | Can’t tell | yes | yes | yes | yes | good |
| Biruk K. &Abetu E. | yes | yes | yes | yes | yes | yes | yes | yes | yes | yes | good |
| Steege R et al | yes | yes | yes | Can’t tell:  questionnaire validation not included | yes | No:  convenient sampling | yes | yes | yes | yes | good |
| Shiferaw S | yes | yes | yes | yes | yes | yes | yes | yes | yes | yes | good |
| Harding K et al | yes | yes | yes | Can’t tell | yes | yes | yes | yes | yes | yes | good |
| Gashu KD et al | yes | yes | yes | yes | yes | yes | yes | yes | yes | yes | good |
